# Molecular mechanism of propofol in reducing kidney injury induced by autologous orthotopic liver transplantation

**DOI:** 10.3389/fimmu.2026.1752787

**Published:** 2026-04-20

**Authors:** Gang Jin, Xiaolin Tang, Beibei Gu, Jiaojie Zhou

**Affiliations:** Department of Anesthesiology, Sichuan Provincial People’s Hospital, School of Medicine, University of Electronic Science and Technology of China, Chengdu, China

**Keywords:** AOLT, ASC, FoxO3a, kidney injury, propofol, pyroptosis, renal tubular epithelial cell, SIRT1

## Abstract

**Objective:**

This study explores the mechanism of propofol in autologous orthotopic liver transplantation (AOLT)-insulted kidney injury.

**Methods:**

A rat model of OALT-induced kidney injury was established, followed by intraperitoneal injection of propofol. Rat renal tubular epithelial cells (RTECs) were induced by hypoxia/reoxygenation. Renal pathological changes were observed via H&E and PAS staining. TNF-α, IL-8, IL-6, IL-1β, and IL-18 levels were measured via ELISA. NLRP3, cleaved Caspase-1, and GSDMD-N expressions were tested via Western blot. The binding of SIRT1 to FOXO3a, SIRT1 to ASC, and ASC to NLRP3, as well as acetylation levels of FOXO3a, ASC, and NLRP3 were analyzed via immunoprecipitation. The binding between FOXO3a and ASC was verified via Ch-IP and dual luciferase assays.

**Results:**

SIRT1 expression is augmented in rat kidney tissues following AOLT, and propofol further elevates SIRT1 expression. Propofol alleviates AOLT-induced kidney injury and pyroptosis. Mechanistically, SIRT1 binds to FOXO3a in the nucleus to inhibit its acetylation, thereby reducing ASC expression. Moreover, SIRT1 binds to ASC in the cytoplasm to inhibit its acetylation, thereby inhibiting the binding of ASC to NLRP3.

**Conclusion:**

In conclusion, Propofol promotes SIRT1-mediated deacetylation to inhibit RTEC pyroptosis via FOXO3a/ASC/NLRP3 axis and alleviate AOLT-induced kidney injury.

## Introduction

Liver transplantation is deemed the most effectual strategy for end-stage liver diseases. However, acute kidney injury (AKI) can result in high perioperative mortality in patients receiving orthotopic liver transplantation (OLT) (1). Therefore, it is crucial to improve the survival and rehabilitation post OLT by controlling or avoiding AKI during the perioperative period of liver transplantation. Notably, the death of renal tubular epithelial cells (RTECs) is not only as a result of AKI, but also a vital pathogenic factor in the development of AKI, as irreversible injury and death of RTECs directly hinders renal function recovery and eventuates poor prognosis (2, 3). Pyroptosis, a programmed cell death modality actuated by inflammasomes and mediated by gasdermin proteins, characterized by plasma membrane swelling and rupture, inflammatory cytokine release, and inflammatory cell infiltration, which is also a special form of death in RTECs (4, 5). Revealing the potential mechanism of RTEC death has important clinical value for elucidating the pathogenesis of AKI post OLT and developing novel effective therapeutic strategies.

Propofol is a short-acting intravenous anaesthetic agent extensively used in clinically. In addition to its anaesthetic advantage, propofol exerts immunomodulatory, anti-inflammatory, antioxidative, and neuroprotective effects (6). Total intravenous anesthesia with propofol reduces the incidence of AKI post lung transplantation by attenuating inflammatory responses (7). Propofol alleviates AKI post OLT by repressing reperfusion-induced enhancement of connexin32 (8). Intravenous injection of propofol can protect liver cells from apoptosis induced by reactive oxygen species (ROS) during liver transplantation (9). Propofol depressed pyroptosis and mitigates renal ischemia/reperfusion (rI/R)-eventuated acute lung injury by activating SIRT1 (10). Moreover, propofol combats pyroptosis of RTECs in the context of rI/R injury (11). Nevertheless, the molecular mechanism of propofol in reducing pyroptosis of RTECs post autologous orthotopic liver transplantation (AOLT) has not been clarified.

Sirtuin 1 (SIRT1), a class III histone/protein deacetylase, is profoundly implicated in various physiological processes including cellular senescence, inflammation, and stress resistance (12). SIRT1 has been established as a crucial conveyer of the anti-pyroptotic effect of propofol during rI/R-insulted acute lung injury (10). SIRT1 inhibits pyroptosis of lipopolysaccharide-stimulated HK-2 cells by mediating deacetylation of Forkhead transcription factor O3a (FOXO3a) (13). In NRK-52E cells, upregulation of nuclear SIRT1 expression decreases FoxO3a acetylation, thereby restraining apoptosis and alleviating AKI post AOLT (14). Accordingly, it is speculated that SIRT1-mediated deacetylation of FOXO3a counteracts pyroptosis of RTECs in AOLT-induced AKI.

The transcription factor FOXO3a manipulates gene expression to partake diverse biological processes including aging, inflammation, oxidative stress, and cell death (15). FOXO3a is regulated by post-translational modifications such as phosphorylation, acetylation, and ubiquitination, each of which affects the transcriptional activity of FOXO3a protein (16). Nuclear FOXO3a can be deacetylated by SIRT1, and Deacetylated FOXO3a is translocated into the nucleus, where it plays a transcriptional role (17). We predict through the JASPAR database that FOXO3a can bind to ASC promoter. ASC is the vital adaptor protein essential for the assembly and activation of canonical inflammasomes (18). NLRP3 inflammasome is the most well-characterized so far. ASC deficiency significantly curbs inflammatory responses and relieves subsequent kidney injury and fibrosis (19). ASC deletion minimizes kidney injury, reduces inflammatory cytokines, protects against apoptosis of tubular cells in unilateral ureter obstruction-induced in mice (20). Moreover, it has been reported that SIRT1 interacts with ASC to reduce its protein stability (21). Another family member of sirtuins, SIRT6, deacetylates ASC to repress its interaction with NLRP3 inflammasome and then restrain pyroptosis of endothelial cells, thus decelerating atherosclerosis progression (22). Based on the above findings, we speculate that propofol reduces FOXO3a acetylation and inhibits ASC expression by promoting SIRT1 into the nucleus, and SIRT1 removes ASC acetylation to inhibit the binding of ASC to NLRP3, thereby reducing pyroptosis of RTECs. In the present work, we focus on the mechanism of propofol in pyroptosis of RTECs. Different from the conventional ischemia model, we applied the AOLT model to replicate the complex scene of kidney transplantation, which involves not only ischemia-reperfusion, but also surgical trauma. This distinction is crucial because the pathophysiology during transplantation may involve more complex microcirculation disorders. This study proves that the protective effect mediated by SIRT1 is effective in this specific transplantation model, and provides a stronger translational medicine basis for the use of propofol during the perioperative period.

## Materials and methods

### Ethics statement

Animal experimental procedures were approved by the Animal Ethics Committee of Sichuan Provincial People’s Hospital (Approval Number:2024-sc74) and implemented following the Guide for the Care and Use of Laboratory Animals.

### Experimental animals

Male Sprague-Dawley rats (200-220 g; Beijing Vital River Laboratory Animal Technology Co., Ltd.) were maintained under standard conditions (25-27 °C, 12/12 h light/dark cycles, SPF laboratory diet and distilled water) for one week before the experimentation.

### Establishment of AOLT model

Three days before AOLT, rats were injected with propofol (Sigma-Aldrich, St. Louis, MO, USA) intraperitoneally at a dose of 50 mg/kg per day (23). This dose of propofol has produced beneficial effects in rats. AOLT modeling was conducted by a researcher who was unaware of the animal grouping, referring to the methods in previous studies (14, 23). In short, rats were fasted for 8 h, but were free to drink water. Under inhalation anesthesia (isoflurane 1-2%), surgery was performed on rats. After entering the abdominal cavity, the hepatic falciform ligament was removed and ligated, and the left diaphragmatic vein along the esophagus was severed. The liver was exposed until the superior vena cava (SVC) was released, and then returned to its original position. A bold line was prepared to for easy guidance of SVC blockage using vascular forceps in the later stage. After the liberation of the left renal vein upper region, the inferior vena cava (IVC) was dissociated and the first hepatic portal vein was dissected for separating the portal vein (PV) from the confluence of the mesenteric and splenic veins. Then, the liberation of the hepatic artery and biliary tract was performed separately in line with their anatomic relationship. Subsequently, we ligated the portal vein and used microvascular forceps at the confluence of the mesenteric vein, splenic vein, hepatic artery, SVC, and IVC. The PV was punctured with a 24-gauge needle for reperfusion. We made a 1-mm incision on the IVC wall as the outflow port and injected 4 °C Ringer lactate solution at a rate of 2.5 mL/min until the liver color turned yellow. We then used an 8–0 needle to close the openings of PV and IVC. PV, SVC, IVC, and hepatic artery were all unclamped. On average, the anhepatic phase lasted for 20 ± 1 min. This surgery simulated the pathology and surgical process during human liver transplantation, including clamping and releasing of the IVC, PV, and hepatic artery, hypoxia, intestinal congestion, as well as liver and kidney I/R injury. Interrupting IVC blood flow during the anhepatic phase can lead to severe systemic hypotension, thus resulting in blood reflux and ultimately kidney ischemia. Rats were euthanized 8 h after modeling (200 mg/kg pentobarbital sodium via intraperitoneal injection), as postoperative AKI was most severe at this time. Kidney samples were collected for histopathological, immunohistochemical, immunofluorescence, and Western blot. In addition, blood was collected from the abdominal aorta for measuring serum creatinine (SCr) and blood urea nitrogen (BUN).

### Experimental grouping

The 48 experimental animals were randomly grouped according to body weight, with 8 animals in each group. Sham group underwent open abdominal vascular dissection without AOLT, and 2 mL of physiological saline was injected intraperitoneally daily for 3 days before surgery; AOLT group underwent AOLT surgery, with 2 mL of physiological saline injected intraperitoneally daily for 3 days prior to the surgery; AOLT + fat milk group received a daily intraperitoneal injection of 2 mL/day fat milk (lipid emulsion vehicle; Mitsubishi Pharma Co., Ltd, Guangzhou, China) 3 days before the AOLT, with the last injection 12 h before the AOLT; AOLT + Pro group received intraperitoneal injection of 50 mg/kg propofol daily for 3 days prior to AOLT, with the last injection occurring 12 h prior to the surgery; AOLT + Pro + AAV9 sh-NC group received intravenous injection of AAV9 virus-packaged sh-NC two weeks before the AOLT, and 50 mg/kg propofol was injected intraperitoneally daily 3 days before the surgery, with the last injection 12 h before the AOLT surgery; AOLT + Pro + AAV9-sh-SIRT1 group received intravenous injection of AAV9 virus-packaged sh-SIRT1 two weeks before the AOLT surgery, and 50 mg/kg propofol was injected intraperitoneally daily 3 days before the surgery, with the last injection given 12 h before the AOLT surgery. Adeno-associated virus 9-mediated SIRT1 interference (AAV9-sh-SIRT1) and its control were obtained from Shanghai JUVENTAS Biotechnology Co., Ltd. (Shanghai, China).

### Histopathological examination

Periodic acid-schiff (PAS) and hematoxylin & eosin (H&E) staining were used to evaluate kidney pathology. Kidney tissue sections were fixed in 10% formalin for approximately 24 h before observation under an optical microscope (Olympus, Tokyo, Japan). According to the percentage of cortical tubular necrosis, the tubular injury in 10 fields of view per group was evaluated at 200x magnification by two pathologists who were blinded to the experiment: 0 = no injury, 1 = 1-25%, 2 = 26-50%,3 = 51-75%, 4 = 76-100%.

### Cell culture and treatment

NRK-52E cells procured from American Type Culture Collection (Manassas, VA, USA) were cultured in DMEM comprising 10% fetal bovine serum at 37°C with 5% CO_2_. To stabilize transduction, cells were infected with AAV9-sh-SIRT1, with AAV9-sh-NC (sh-NC) as the control. Puromycin or Hygromycin B was used to screen for NRK-52E cells with stable expression.

To induce hypoxia/reoxygenation (H/R) injury, NRK-52E cells were cultured in serum-free and glucose-free medium in a moist oxygen-deficient environment (95% N_2_ + 5% CO_2_) for 24 h. For reoxygenation, cells were stored under normal oxygen environment (95% air+5% CO_2_) for 4 h. During hypoxia, cells were treated with 15 μM propofol (23) for 24 h. After processing, cells and supernatant were collected separately for analysis. The control cells were kept in 95% air + 5% CO_2_ for 28 h.

### Immunofluorescence

Immunofluorescence was performed on 5 μm kidney sections embedded in paraffin and NRK-52E cells treated with different methods. Antibodies against Caspase-1 (1:100, MA5-16215, Invitrogen, Carlsbad, CA, USA) and SIRT1 (1:100, ab189494, Abcam, Cambridge, MA, USA) were used. The cell nucleus was stained with 4’,6-diamidino-2-phenylindole (DAPI). All images were captured by a fluorescence microscope (EVOS FL, Life Technology). Animal experiments were performed with 8 animals per group, and cell experiments were conducted in three biological replicates.

### Determination of physiological and biochemical indicators

Renal function was evaluated by measuring BUN and Scr levels. Blood samples were collected 8 h after AOLT surgery, and serum was separated by centrifugation. BUN and Scr levels in serum were measured by an automated biochemical analyzer (Chemray 240, Shenzhen, China). Animal experiments were performed with 8 animals per group.

Malondialdehyde (MDA) content was detected using an MDA assay kit (KGA7101-100; KeyGen Biotech Co., Ltd., Jiangsu, China). Superoxide dismutase (SOD) activity was tested strictly following the instruction of the detection kit (BC0170, Solarbio, Beijing, China). Animal experiments were performed with 8 animals per group, and cell experiments were conducted in three biological replicates.

### Reactive oxygen species detection

ROS activity was measured using ROS assay kit (Servicebio, Wuhan, China). Frozen kidney sections were stained with 10 μM dihydroetorphine (DHE) (Servicebio) at 37°C for 30 min, and the percentage of DHE stained area was observed under a confocal microscopy. Animal experiments were performed with 8 animals per group.

### ELISA

After washing with pre-cooled phosphate buffer saline (PBS), the cut tissues were mixed with an equal amount of PBS (1:9) on ice and centrifuged at 4 °C for 10 min at 5000 g. After centrifugation (1000 g, 4 °C for 10 min), the culture supernatant was obtained and diluted to 1:3. The levels of TNF-α (MBS282960, MyBiosource, San Diego, CA, USA), IL-6 (MBS2021530, MyBiosource), IL-1β (MBS2023030, MyBiosource), and IL-18 (MBS2021706, MyBiosource) in tissues and cells were detected using commercially available ELISA kits. Animal experiments were performed with 8 animals per group, and cell experiments were conducted in three biological replicates.

### Western blot

Cells and kidney tissues were stored at -80°C. Proteins were extracted from samples using radio-immunoprecipitation assay (RIPA) lysis buffer (Beyotime, Shanghai, China), subjected to 8%-12% SDS-PAGE, and transferred onto PVDF membranes. After blocking treatment, the membranes were treated with primary antibodies overnight including NLRP3 (1:1000, ab263899, Abcam), cleaved Caspase-1 (1:1000, PA5-99390, Invitrogen), GSDMD-N (1:2000, YM8489, Immunoway), SIRT1 (1:1000, ab189494, Abcam), FOXO3a (1:1000, MA5-14932, Invitrogen), ASC (1:1000, ab309497, Abcam), β-actin (1:1000, ab8227, Abcam), Histone H3 (1:2000, ab1791, Abcam), and acetylated lysine (1:1000, MA5-33031, Invitrogen), followed by incubation with IgG (1:5000, ab6721, Abcam) to recognize immune response bands. The bands were detected using enhanced chemiluminescence assay kit (Thermo Fisher Scientific Inc.). Animal experiments were performed with 8 animals per group, and cell experiments were conducted in three biological replicates.

### RT-qPCR

TRIzol reagent (Invitrogen) was exploited for total RNA extraction, and cDNA was generated using HyperScript III RT SuperMix for qPCR and gDNA Remover (EnzyArtisan, Shanghai, China). NovoStart^®^SYBR qPCR SuperMix Plus (Novoprotein, Shanghai, China) was employed to prepare qPCR system. The relative expression of target gene was calculated by the 2^-△△Ct^ method (24), with GAPDH as the internal reference. Primers are shown in **Table 1**. Animal experiments were performed with 8 animals per group, and cell experiments were conducted in three biological replicates.

**Table 1:** PCR primers.

Name	Sequence (5’-3’)
SIRT1	F: TTCAAGGCTGTTGGTTCCAG
	R: AGGCCAGCATTTTCTCACTG
FOXO3a	F: TTGGTTTGAACGTGGGGAAC
	R: AGTTTGAGGGTCTGCTTTGC
ASC promoter	F: GTCCAGATCTGGTTTCTGCTC
	R: CTGGTCAAACCTAGGGCCCCA
ASC	F: TGATGGTTTGCTGGATGCTC
	R: TTTTGGTTGGTGGTCTCTGC
GAPDH	F: AACTTTGGCATCGTGGAAGG
	R: GTGGATGCAGGGATGATGTTC

SIRT1, sirtuin 1; FOXO3a, forkhead box O3A; ASC, apoptosis associated speck-like protein containing a CARD; GAPDH, glyceraldehyde-3-phosphate dehydrogenase.

### Cell counting kit-8 assay

NRK-52E cells seeded in a 96-well plate (3 × 10^3^) were cultured for 48 h. Each well was treated with 10 µL of CCK-8 solution (Dojindo, Tokyo, Japan) at 37°C for 2 h. Finally, the absorbance value at 450 nm was measured by a microplate reader. Cell experiments were conducted in three biological replicates.

### Flow cytometry

Cell pyroptosis was assessed using FAM-FLICA Caspase-1 assay kit (ICT, USA). Cells were washed, suspended in PBS (pH = 7.4), and stained with FLICA/PI. Pyroptotic cells was quantified by a flow cytometer (BD Biosciences, San Jose, USA). Cells showing positive staining for both dyes were deemed pyroptotic cells. Cell experiments were conducted in three biological replicates.

### Nuclear cytoplasmic separation

The nuclear protein was isolated and purified using Nuclear and Cytoplasmic Extraction Kit (BestBio, Shanghai, China). Nuclear and cytoplasmic proteins were calculated by immunoblotting. According to the standardized nuclear protein content of histone H3, the cytoplasmic protein content was standardized by β-actin. Cell experiments were conducted in three biological replicates.

### Co-immunoprecipitation

Cells or kidney tissues were lysed in RIPA buffer and centrifuged at 12000 g for 15 min to obtain the soluble fraction. An equal amount of soluble fraction was treated with anti-SIRT1 (1:30, ab189494, Abcam), ASC (1:30, ab309497, Abcam), NLRP3 (1:30, ab263899, Abcam), and FOXO3a (1:100, MA5-14932, Invitrogen) antibodies, and then bound to protein A/G agarose beads to generate immune complexes. Finally, the binding protein was eluted by boiling with sample buffer, followed by Western blot analysis. Animal experiments were performed with 8 animals per group, and cell experiments were conducted in three biological replicates.

### Chromatin immunoprecipitation

Cells were incubated with 1% formaldehyde at room temperature for 10 min for cross-linking. Next, cells were lysed with lysis buffer and sonicated for 30 min. The supernatant was incubated with anti-FOXO3a (MA5-14932, Invitrogen), with rabbit IgG (MA5-56524, Invitrogen) as a control. Immunoprecipitated DNA was identified via qRT-PCR. **Table 1** displays primers. Animal experiments were performed with 8 animals per group, and cell experiments were conducted in three biological replicates.

### Dual luciferase reporter assay

ASC promoter region containing wild-type (ASC-WT) or mutant-type (ASC-MUT) FOXO3a binding site was cloned into pGL3-basic vector. Cells were co-transfected with luciferase vector and designated plasmid for 48 h. Afterwards, the dual luciferase reporter gene detection system (Promega, Madison, Wisconsin, USA) was applied to measure luciferase activity. FOXO3a overexpression vector (oe FOXO3a) and empty vector (oe NC) were synthesized by Shanghai Heyuan Biotechnology Co., Ltd. Cell experiments were conducted in three biological replicates.

### Statistical analysis

SPSS 21.0 (IBM Corp., Armonk, NY, USA) and GraphPad Prism 8.0 (GraphPad Software Inc., San Diego, CA, USA) were applied for data analysis and map plotting. The data were in normal distribution with uniform variance. Pearson chi square test or Fisher’s exact test was exploited to two-group comparisons of counting data, while t-test was used for two-group comparisons of measurement data. One-way or two-way analysis of variance (ANOVA) was employed for multi-group comparisons, followed by Tukey’s multiple comparison test. *P* < 0.05 indicated a significant difference.

## Results

### Propofol alleviates kidney injury induced by AOLT

**Figure 1:**
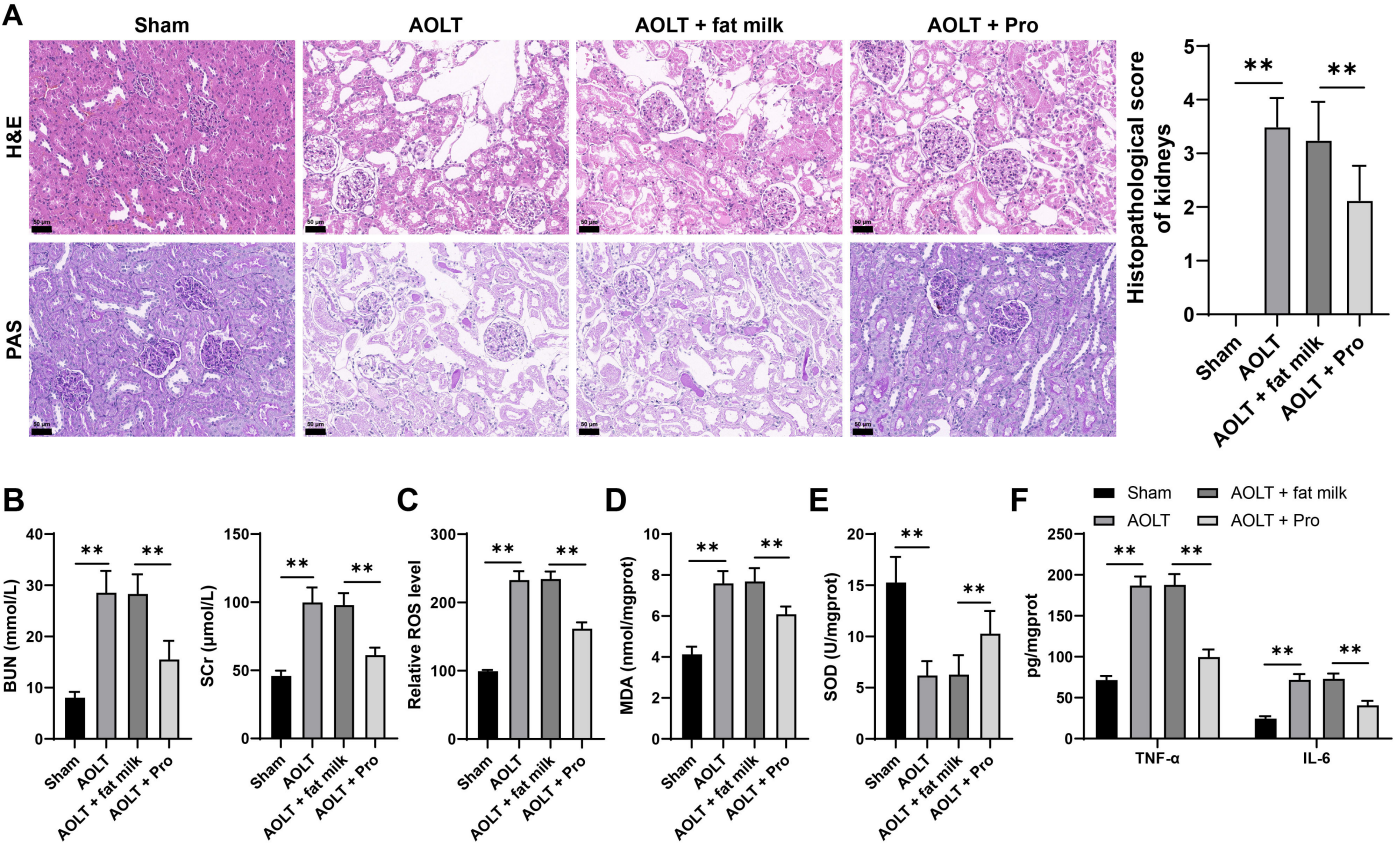
Propofol alleviates kidney injury induced by AOLT. A rat model of AOLT induced kidney injury was established through surgery. Three days before AOLT surgery, rats were intraperitoneally injected with propofol daily, with fat milk as the control. **(A)** Observation of pathological changes in renal tissues by H&E and PAS staining, n = 8; **(B)** Serum BUN and Scr levels, n = 8; **(C–E)** ROS **(C)**, MDA **(D)**, and SOD **(E)** levels in renal tissues, n = 8; **(F)** TNF-α and IL-6 levels in renal tissues detected by ELISA, n = 8. Data are displayed as mean ± SD. Data in panels **(A–E)** were analyzed by one-way ANOVA, and data in panel **(F)** were analyzed by two-way ANOVA, followed by Tukey’s multiple comparisons test. **P < 0.01.

A rat model of AOLT was induced to explore whether propofol was beneficial in alleviating AOLT induced kidney injury. Eight hours after AOLT surgery, we observed severe renal tissue injury in rats through H&E and PAS staining (P<0.01, **Figure 1A**), and elevated serum BUN and Scr levels (P<0.01, **Figure 1B**). ROS and MDA levels in renal tissues were increased, while SOD levels were decreased (P<0.01, **Figures 1C–E**), indicating an increase in oxidative stress in renal tissues. ELISA unveiled that inflammatory factors (TNF-α, IL-6) were dramatically increased (P<0.01, **Figure 1F**). The above phenomena were alleviated to a certain extent after injection of propofol (P<0.01, **Figures 1A–F**) These results indicate that propofol can alleviate kidney injury caused by AOLT.

### Propofol alleviates cell pyroptosis in renal tissues caused by AOLT

**Figure 2:**
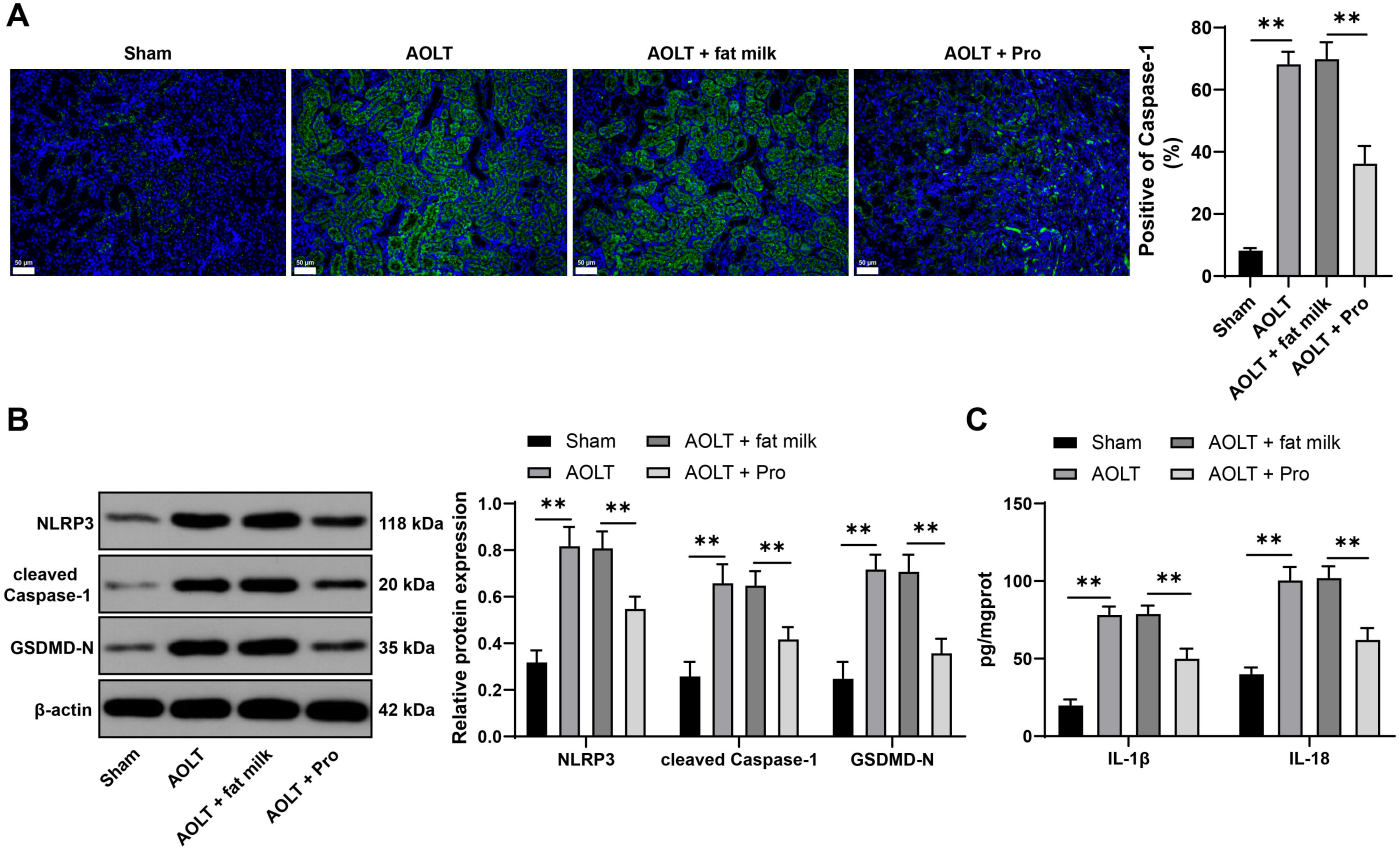
Propofol alleviates cell pyroptosis in renal tissues caused by AOLT. A rat model of AOLT induced kidney injury was established through surgery. Three days before AOLT surgery, rats were intraperitoneally injected with propofol daily, with fat milk as the control. **(A)** Caspase-1 expression in renal tissues detected by immunofluorescence, n = 8; **(B)** NLRP3, cleaved Caspase-1, and GSDMD-N expression in renal tissues detected by Western blot, n = 8; **(C)** IL-1β and IL-18 levels in renal tissues detected by ELISA, n = 8. Data are displayed as mean ± SD. Data in **(A)** were analyzed by one-way ANOVA, and data in **(B, C)** were analyzed by two-way ANOVA, followed by Tukey’s multiple comparisons test, **P < 0.01.

Furthermore, we observed the changes in cell pyroptosis levels in renal tissues. Immunofluorescence displayed that after AOLT, the fluorescence of Caspase-1 in renal tissues was enhanced, while the fluorescence was weakened after propofol treatment (P<0.01, **Figure 2A**). NLRP3, cleaved Caspase-1, GSDMD-N, and pyroptosis related inflammatory factors (IL-1β, IL-18) were significantly elevated after AOLT (P<0.01, **Figures 2B, C**), while propofol reduced the levels of these factors (P<0.01, **Figures 2A–C**). These results imply that propofol alleviates cell pyroptosis in renal tissues caused by AOLT.

### Propofol reduces renal tubular epithelial cell pyroptosis *in vitro*

**Figure 3:**
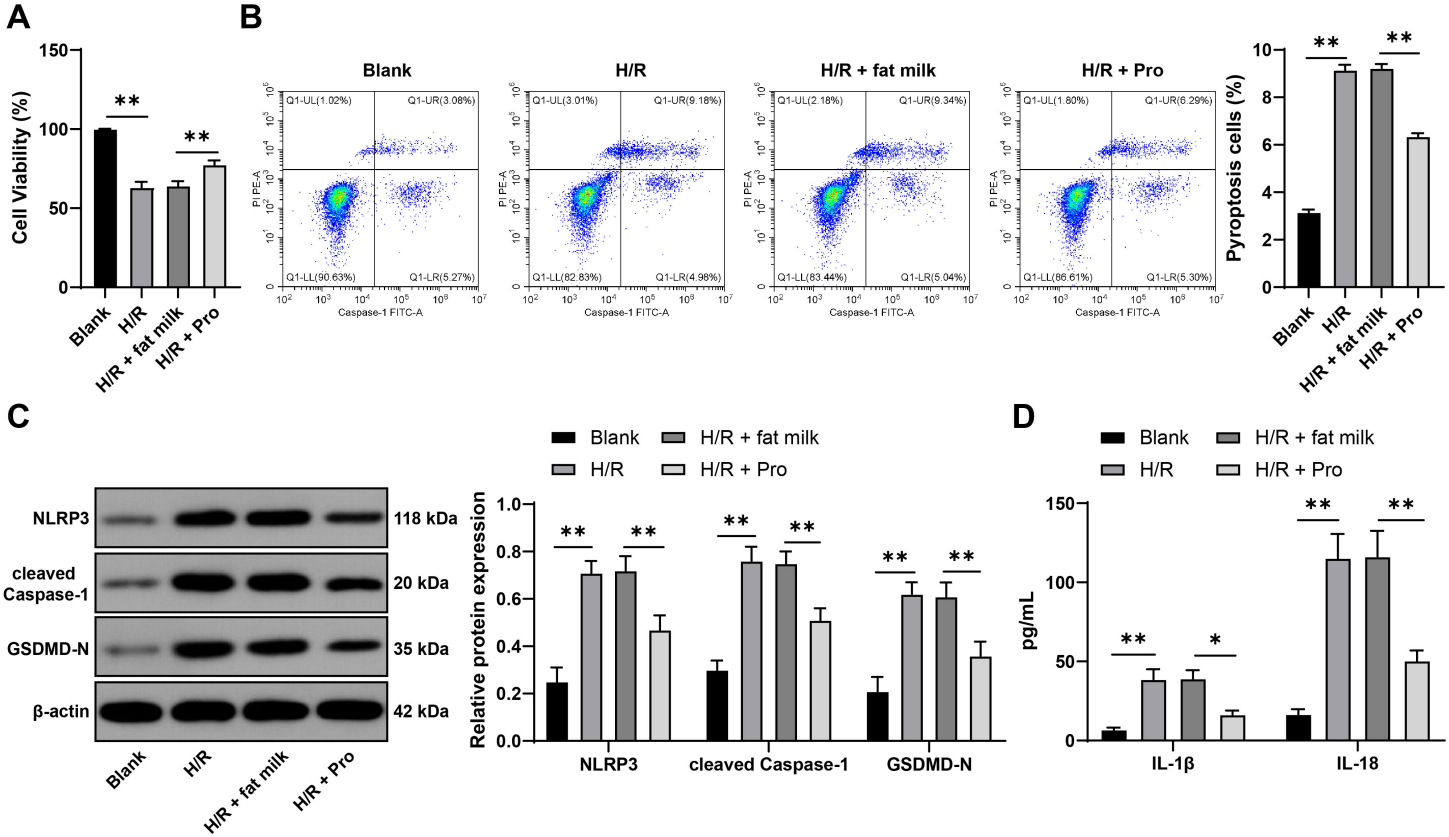
Propofol reduces renal tubular epithelial cell pyroptosis *in vitro.* NRK-52E cells were induced by H/R to simulate the AOLT process and treated with propofol, with fat milk as the control. **(A)** Cell viability detected by CCK-8 assay; **(B)** Cell pyroptosis detected by flow cytometry, n = 3; **(C)** NLRP3, cleaved Caspase-1, GSDMD-N expression in cells detected by Western blot, n = 3; **(D)** IL-1β and IL-18 levels in cells detected by ELISA, n = 3. Data are displayed as mean ± SD. Data in **(A, B)** were analyzed by one-way ANOVA, and data in **(C, D)** were analyzed by two-way ANOVA, followed by Tukey’s multiple comparisons test, *P < 0.05, **P < 0.01.

To further explore the role of propofol in cell pyroptosis, we cultured NRK-52E cells *in vitro* and simulated the AOLT process by H/R, followed by propofol treatment. H/R abated the NRK-52E cell viability (P<0.01, **Figure 3A**), augmented pyroptotic cells (P<0.01, **Figure 3B**), uplift NLRP3, cleaved Caspase-1, and GSDMD-N (P<0.01, **Figure 3C**), and upregulated IL-1β and IL-18 (P<0.01, **Figure 3D**). Propofol treatment enhanced cell viability (P<0.01, **Figure 3A**), reduced cell pyroptosis (P<0.01, **Figure 3B**), decreased NLRP3, cleaved Caspase-1 and GSDMD-N (P<0.01, **Figure 3C**), and diminished IL-1β and IL-18 (P<0.05, **Figure 3D**). Briefly, propofol can also alleviate renal tubular epithelial cell pyroptosis *in vitro*.

### Propofol promotes SIRT1 expression, while nuclear SIRT1 inhibits ASC expression by removing FOXO3a acetylation

**Figure 4:**
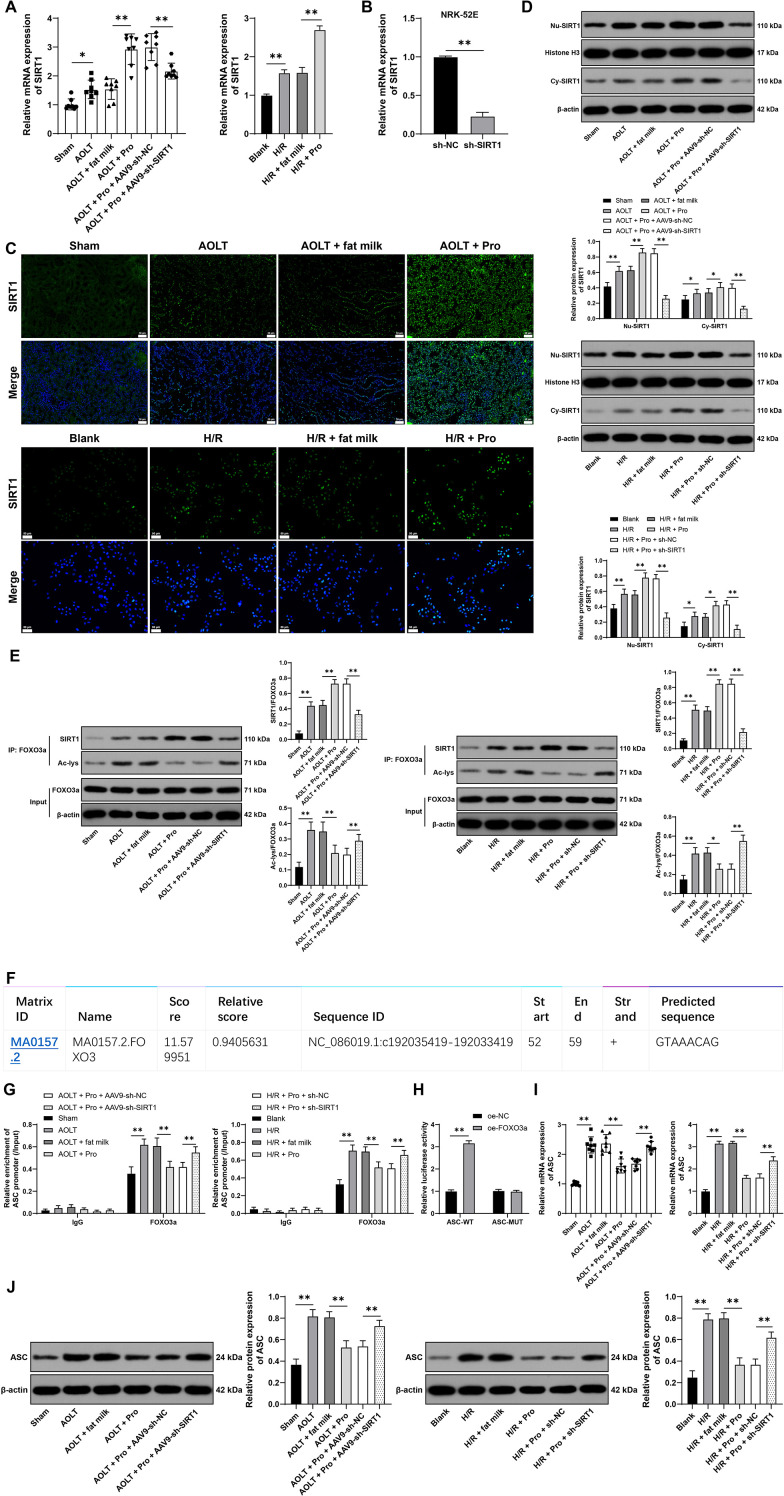
Propofol promotes SIRT1 expression, while nuclear SIRT1 inhibits ASC expression by removing FOXO3a acetylation. **(A)** SIRT1 expression in renal tissues (n = 8) and cells (n = 3) detected by qRT-PCR; **(B)** NRK-52E cells were infected with AAV9-sh-SIRT1 (sh-SIRT1), with AAV9-sh-NC (sh-NC) as a control. SIRT1 expression in cells detected by qRT-PCR, n = 3; **(C)** Observation of the localization of SIRT1 in renal tissues (n = 8) and cells (n = 3) by immunofluorescence, DAPI (blue) representing the nucleus; **(D)** After nuclear cytoplasmic separation, detection of SIRT1 expression in the cytoplasm (Cy-) and nucleus (Nu-) of renal tissues (n = 8) and cells (n = 3) by Western blot, respectively, with Histone H3 as the internal reference for nuclear proteins, and β-actin as the internal reference for cytoplasmic proteins; **(E)** Immunoprecipitation detection of SIRT1 binding to FOXO3a and acetylation levels of FOXO3a in renal tissues (n = 8) and cells (n = 3); **(F)** Prediction of the binding site of FOXO3 to ASC promoter through the JASPAR database; **(G)** ChIP analysis of FOXO3a enrichment on ASC promoter in tissues (n = 8) and cells (n = 3); **(H)** Validation of the binding relationship between FOXO3a and ASC promoter by dual luciferase reporter assay, n = 3; **(I, J)** Detection of ASC expression in tissues (n = 8) and cells (n = 3) by qRT-PCR and Western blot. Data are displayed as mean ± SD. Data in **(B)** were analyzed by t test. Data in **(A, I, J)** were analyzed by one-way ANOVA, and data in **(D, E, G, H)** were analyzed by two-way ANOVA, followed by Tukey’s multiple comparisons test, *P < 0.05, **P < 0.01.

After AOLT and H/R, SIRT1 expression in renal tissues and cells was significantly uplift (P<0.05, **Figures 4A, D**). Propofol further boosted SIRT1 expression (P<0.05, **Figures 4A, D**) Immunofluorescence and nuclear cytoplasmic separation assay results showed that propofol augmented SIRT1 expression in the nucleus and cytoplasm (P<0.05, **Figures 4C, D**), with a more notable surge in nuclear SIRT1 expression (P<0.05, **Figure 4D**). Nuclear SIRT1 inhibits AOLT-induced renal cell apoptosis by suppressing FOXO3a acetylation (14). We speculated that propofol was involved in cell pyroptosis by regulating SIRT1 to remove FOXO3a acetylation. After propofol treatment, the binding of SIRT1 to FOXO3a was increased, but the acetylation level of FOXO3a was significantly declined (P<0.01, **Figure 4E**). To verify that propofol regulated the acetylation of FOXO3a through SIRT1, we inhibited SIRT1 expression in rats and cells (P<0.01, **Figures 4A, B, D**), following propofol treatment. It was found that inhibition of SIRT1 did not affect FOXO3a expression but significantly elevated acetylation level of FOXO3a (P<0.01, **Figure 4E**). JASPAR database prediction showed that FOXO3a had a binding site with the ASC promoter (**Figure 4F**). Ch-IP analysis showed that the enrichment of FOXO3a on the ASC promoter was reduced after propofol treatment but increased after inhibition of SIRT1 (P<0.01, **Figure 4G**). Dual luciferase reporter assay unraveled that overexpression of FOXO3a significantly increased luciferase activity in the ASC-WT group (P<0.01, **Figure 4H**). ASC was highly expressed in renal tissues and cells after AOLT and H/R (P<0.01, **Figures 4I, J**). Propofol treatment decreased ASC expression, while inhibition of SIRT1 expression increased ASC expression (P<0.01, **Figures 4I, J**). Shortly, propofol promotes SIRT1 expression, while nuclear SIRT1 inhibits the transcriptional promotion of ASC by removing FOXO3a acetylation, thereby suppressing ASC expression.

### Cytoplasmic SIRT1 inhibits the interaction between ASC and NLRP3 through deacetylation

**Figure 5:**
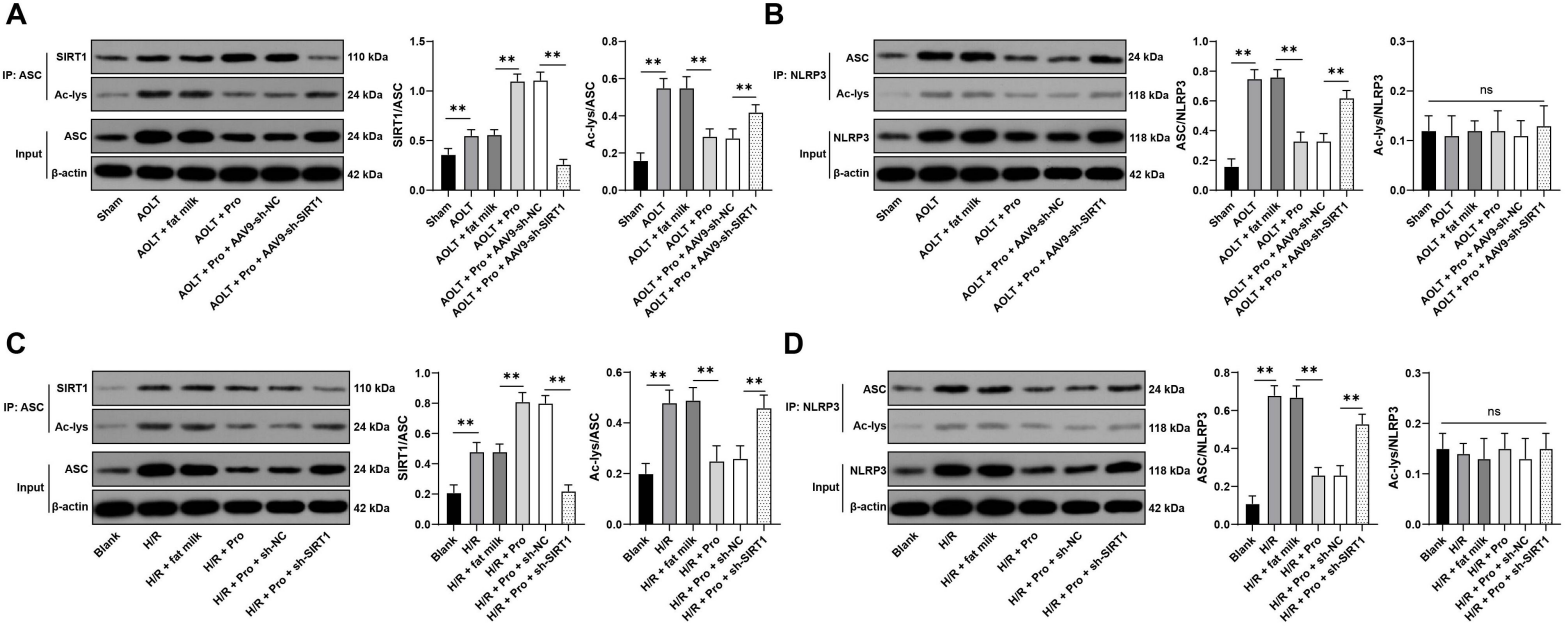
Cytoplasmic SIRT1 inhibits the interaction between ASC and NLRP3 through deacetylation. **(A–D)** Immunoprecipitation detection of the binding between SIRT1 and ASC in tissues (n = 8) and cells (n = 3), the binding between ASC and NLRP3, as well as the acetylation levels of ASC and NLRP3. Data are displayed as mean ± SD and analyzed by one-way ANOVA, followed by Tukey’s multiple comparisons test; ns, *P* > 0.05; **P < 0.01.

Next, we attempted to explore whether cytoplasmic SIRT1 was also involved in cell pyroptosis. SIRT6 reduces the deacetylation of ASC, and the binding of ASC to NLRP3 inhibits cell pyroptosis (22). Propofol treatment increased the binding of SIRT1 to ASC (P<0.01, **Figures 5A, C**) and reduced the acetylation level of ASC, while inhibition of SIRT1 augmented the acetylation level of ASC (P<0.01, **Figures 5A, C**) and the acetylation level of NLRP3 was not affected (P>0.05, **Figures 5B, D**). Meanwhile, propofol treatment reduced the binding of ASC to NLRP3 (P<0.01, **Figures 5B, D**). After inhibition of SIRT1, the binding between ASC and NLRP3 was increased (P<0.01, **Figures 5B, D**). Cytoplasmic SIRT1 inhibits the interaction between ASC and NLRP3 through deacetylation.

### Inhibition of SIRT1 expression partially reverses the inhibitory effect of propofol on renal tubular epithelial cell pyroptosis

**Figure 6:**
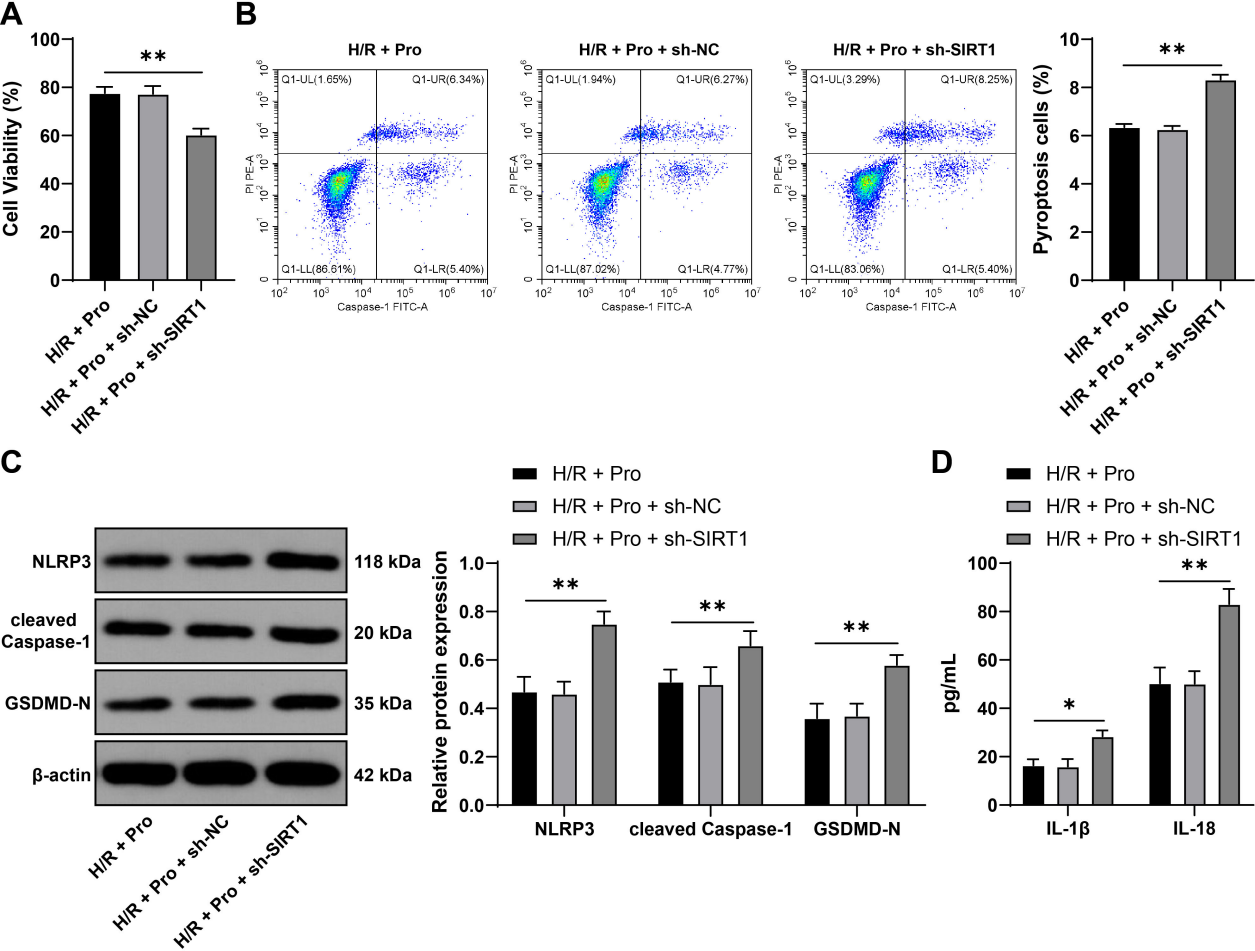
Inhibition of SIRT1 expression partially reverses the inhibitory effect of propofol on renal tubular epithelial cell pyroptosis. NRK-52E cells were infected with AAV9-sh-SIRT1 (sh-SIRT1), with AAV9-sh-NC (sh-NC) as a control, followed by H/R and propofol treatment. **(A)** Cell viability detected by CCK-8 assay, n = 3; **(B)** Cell pyroptosis detected by flow cytometry, n = 3; **(C)** NLRP3, cleaved Caspase-1, GSDMD-N expression in cells detected by Western blot, n = 3; **(D)** IL-1β and IL-18 levels in cells detected by ELISA, n = 3. Data are displayed as mean ± SD. Data in **(A, B)** were analyzed by one-way ANOVA, and data in **(C, D)** were analyzed by two-way ANOVA, followed by Tukey’s multiple comparisons test, *P < 0.05, **P < 0.01.

We inhibited SIRT1 expression *in vitro* and conducted a combined experiment with propofol. Compared with propofol treatment, inhibition of SIRT1 reduced NRK-52E cell viability (P<0.01, **Figure 6A**), elevated pyroptotic cells (P<0.01, **Figure 6B**), uplift NLRP3, cleaved Caspase-1, and GSDMD-N (P<0.01, **Figure 6C**), and boosted IL-1β and IL-18 levels (P<0.05, **Figure 6D**). Inhibition of SIRT1 partially abolishes the inhibitory effect of propofol on renal tubular epithelial cell pyroptosis.

### Inhibition of SIRT1 expression partially reverses the improvement effect of propofol on AOLT induced kidney injury

**Figure 7:**
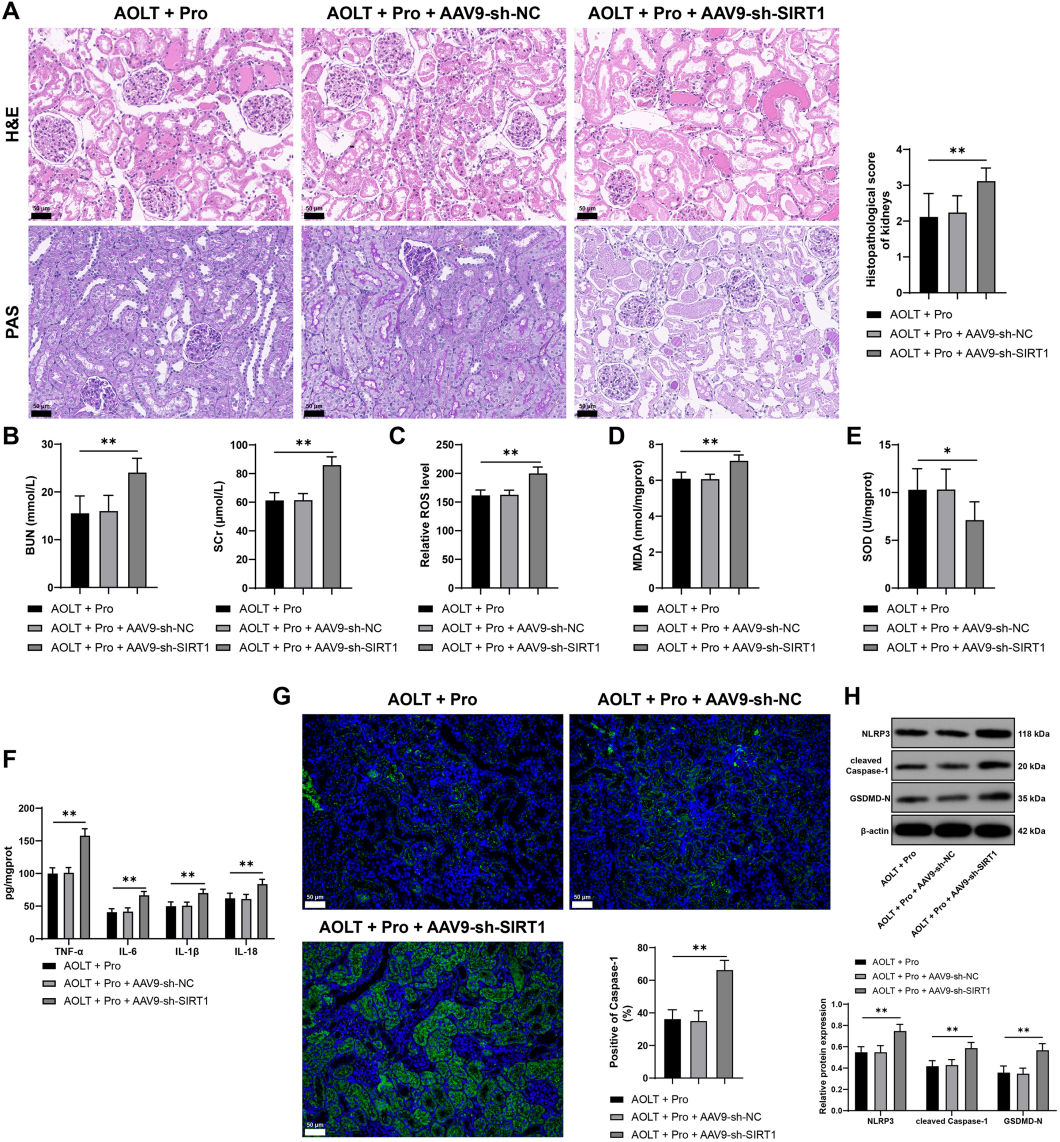
Inhibition of SIRT1 partially reverses the improvement effect of propofol on AOLT induced kidney injury. A rat model of AOLT induced kidney injury was established through surgery. Two weeks before the AOLT surgery, AAV9 virus packaged sh-SIRT1 (AAV9-sh-SIRT1) was injected into rats via the tail vein, with AAV9 sh-NC as the control; propofol was injected intraperitoneally daily for 3 days before surgery, with fat milk as the control. **(A)** Observation of pathological changes in renal tissues by H&E and PAS staining, n = 8; **(B)** Serum BUN and Scr levels, n = 8; **(C–E)** ROS **(C)**, MDA **(D)**, and SOD **(E)** levels in renal tissues, n = 8; **(F)** TNF-α, IL-6, IL-1β, IL-18 levels in renal tissues detected by ELISA, n = 8; **(G)** Caspase-1 expression in renal tissues detected by immunofluorescence, n = 8; **(H)** NLRP3, cleaved Caspase-1, GSDMD-N expression in renal tissues detected by Western blot, n = 8. Data are displayed as mean ± SD. Data in **(A–E, G)** were analyzed by one-way ANOVA, and data in **(F, H)** were analyzed by two-way ANOVA, followed by Tukey’s multiple comparisons test, *P < 0.05, **P < 0.01.

Finally, we reduced the expression of SIRT1 in rats and treated them with propofol. Compared with propofol treatment, reduction of SIRT1 expression aggravated renal tissue injury in rats (P<0.01, **Figure 7A**), increased serum BUN and Scr (P<0.01, **Figure 7B**), elevated ROS and MDA levels in renal tissues, decreased SOD levels (P<0.01, **Figures 7C–E**), increased inflammatory factors (TNF-α, IL-6, IL-1β, IL-18) in renal tissues (*P* < 0.01, **Figure 7F**), and elevated pyroptosis related proteins (NLRP3, cleaved Caspase-1, GSDMD-N) (*P* < 0.01, **Figures 7G, H**). Inhibition of SIRT1 expression counteracts the improvement effect of propofol on AOLT induced kidney injury and cell pyroptosis.

## Discussion

**Figure 8:**
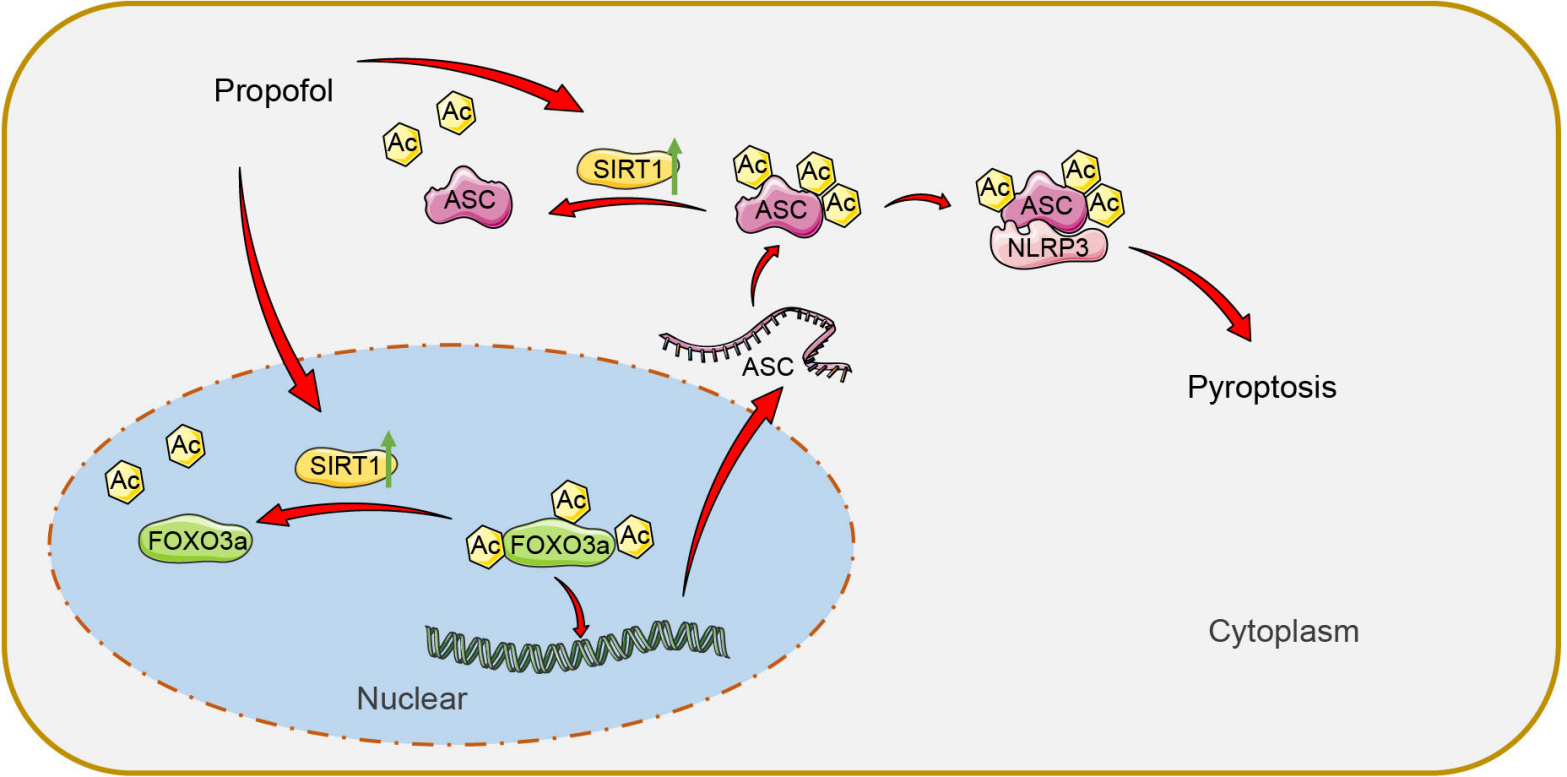
Propofol promotes the expression of SIRT1 to remove the acetylation of nuclear FOXO3a and inhibit the transcriptional promotion effect of FOXO3a on ASC. Meanwhile, SIRT1 removes the acetylation of ASC in the cytoplasm and inhibits its interaction with NLRP3, ultimately inhibiting AOLT induced cell pyroptosis.

Postoperative AKI is a prevalent and severe complication post OLT, which significantly compromises patient survival (1). The death of RTECs is a critical pathology underlying AKI (25). Propofol, as an extensively applied anesthetic, can confer organ protection following liver transplantation (26). This study reveals that propofol promotes the expression of SIRT1 to remove the acetylation of FOXO3a in the nucleus and inhibit the transcriptional promotion of ASC by FOXO3a. Meanwhile, SIRT1 removes the acetylation of ASC in the cytoplasm and inhibits the interaction between ASC and NLRP3, ultimately inhibiting AOLT-induced cell pyroptosis (**Figure 8**).

In clinical and experimental studies, propofol can regulate inflammatory response and oxidative stress caused by organ transplantation surgery stimulation (27). A randomized clinical trial addresses that compared with sevoflurane anesthesia, total intravenous anesthesia with propofol reduces the incidence of AKI following lung transplantation (7). We established an AOLT rat model and observed that propofol injection was beneficial for AOLT-induced kidney injury. Pre-treatment with propofol exerts renal protection against AOLT by uplifting nuclear Nrf2 expression (28). In hypoxia-reoxygenation-induced NRK-52E cells, propofol reduces ROS production and attenuates cell injury by inhibiting Cx32 function (8). In AKI progression, RTECs undergo multiple forms of death, leading to profound pathological changes (25). SIRT1-triggered p65 deacetylation combats pyroptosis and relieves Cadmium-induced chronic kidney injury (29). Propofol also significantly ameliorates pyroptosis of RTECs in the context of renal ischemia/reperfusion via the miR-143-3p/ATP1A2 axis (11). Similarly, we found that propofol alleviated pyroptosis induced by AOLT in rat kidney tissues. We cultured rat RTEC line NRK-52E *in vitro* and simulated the AOLT process through H/R, and found that propofol also reduced pyroptosis of RTECs *in vitro*.

Thereafter, we attempted to investigate the specific mechanism of propofol inhibiting pyroptosis post AOLT. Propofol attenuates pyroptosis in rI/R-insulted acute lung injury by elevating SIRT1 expression in the lungs (10). We observed that SIRT1 expression was notably elevated in kidney tissues of AOLT rat and H/R-induced NRK-52E cells, and propofol further augmented SIRT1 expression. Inhibition of SIRT1 expression *in vitro* and *in vivo* partially counteracted the inhibitory effect of propofol on pyroptosis of RTECs. Immunofluorescence and nuclear cytoplasmic separation analyses showed that propofol boosted SIRT1 expression in the nucleus and cytoplasm, with a more significant surge in nuclear SIRT1 expression. Nuclear SIRT1 expression is elevated in NRK-52E cells following AOLT, which alleviates apoptosis and AKI by promoting FoxO3a deacetylation (14). We speculated that propofol participated in pyroptosis by regulating SIRT1 to remove FOXO3a acetylation. FOXO3a activity can be regulated by post-translational modifications, which alter the subcellular localization of FOXO3a and its DNA binding affinity (16). FOXO3a is activated under metabolic and oxidative stress to mitigate tubular injury and promote cell survival (30). After propofol treatment, the binding of SIRT1 to FOXO3a was increased, but the acetylation level of FOXO3a was dramatically declined. Inhibition of SIRT1 did not impact FOXO3a expression, but its acetylation level was significantly elevated. These results confirmed that propofol regulated acetylation of FOXO3a through SIRT1.

Furthermore, JASPAR database prediction revealed a binding site between FOXO3a and ASC promoter. SIRT1 can also promote the deacetylation of ASC (21). ASC is an adaptor protein that activates various apoptotic and pyroptotic factors by mediating the assembly of signal complexes in apoptotic, inflammatory, and non-inflammatory pathways (31). High expression of ASC in renal collecting duct epithelial cells deteriorates inflammation post unilateral ureteral obstruction (19). ASC deletion relieves unilateral ureteral obstruction-induced renal fibrosis and endoplasmic reticulum stress in mice (20). Our results unveiled that ASC was highly expressed in AOLT rat renal tissues and H/R-induced cells. After treatment with propofol, ASC expression was decreased, but increased after inhibition of SIRT1 expression. The above results indicated that propofol promoted the expression of SIRT1, while nuclear SIRT1 inhibited the transcriptional promotion of ASC by removing FOXO3a acetylation, thereby suppressing ASC expression.

Moreover, we explored whether SIRT1 in the cytoplasm was also involved in the process of cell pyroptosis. A previous report states that SIRT6 deacetylates ASC, thereby reducing the binding of ASC to NLRP3 and inhibiting cell pyroptosis (22). Similarly, we found that propofol treatment increased the binding of SIRT1 to ASC and significantly reduced the acetylation level of ASC. However, inhibition of SIRT1 increased the acetylation level of ASC, while the acetylation level of NLRP3 was not affected. Meanwhile, propofol reduced the binding of ASC to NLRP3, while inhibition of SIRT1 enhanced the binding of ASC to NLRP3. The above results indicated that SIRT1 in the cytoplasm inhibited the binding of ASC to NLRP3 by removing ASC acetylation. It is noteworthy that, given the pharmacokinetic disparities of propofol between humans and rats as different species, the study implemented a preoperative 3-day propofol pretreatment protocol. This approach enables the attainment of drug exposure durations and target regulatory effects in rats that are equivalent to those in humans. Based on the dual regulatory mechanism we have identified, we offer the following hypotheses concerning the potential impacts of intraoperative or postoperative drug administration: 1. A single intraoperative dose may confer partial protection through rapid intracellular mechanisms (where SIRT1 directly deacetylates ASC, thereby inhibiting its interaction with NLRP3). This represents a swift post-translational event that does not necessitate new protein synthesis. 2. Continuous postoperative sedation may sustain SIRT1 activity while concurrently inducing cytoplasmic modifications. If the infusion period is sufficiently prolonged, it could theoretically influence the transcriptional level of ASC, although further investigation is required to substantiate this. 3. Drawing on the principle of allometric scaling, we hypothesize that the combination of an intraoperative loading dose followed by short-term postoperative sedation infusion may represent the most viable strategy for clinical application, as it can not only induce rapid post-translational modifications but also provide the necessary temporal framework for transcriptional regulation.

In conclusion, pyroptosis is one of the important modes of cell death in AKI induced by AOLT. Our study demonstrates that propofol can reduce pyroptosis of RTECs and alleviate kidney injury induced by AOLT. The innovation of our study lies in the discovery that SIRT1 regulates ASC through dual pathways. On the one hand, propofol promotes nuclear SIRT1 expression to remove FOXO3a acetylation and inhibit FOXO3a’s transcriptional promotion on ASC, thereby reducing ASC synthesis. On the other hand, cytoplasmic SIRT1 reduces the binding of ASC to NLRP3 by removing the acetylation of ASC, thereby inhibiting inflammasome assembly and pyroptosis of RTECs. This nuclear-cytoplasmic co-regulatory mode extends the anti-pyroptosis mechanism of SIRT1 from a single modification to the transcriptional regulatory level, and emphasizes the importance of ASC as a key node molecule. To our knowledge, this mechanism of simultaneously regulating ASC expression and function through SIRT1 has not been reported in the field of kidney transplantation or propofol therapy.

However, this study has certain limitations. First of all, the preoperative 3-day propofol pretreatment protocol employed in this study differs from the conventional perioperative propofol administration used in clinical liver transplantation. The findings of this study should be regarded as a conceptual validation of the target’s efficacy, rather than a direct endorsement for clinical dosing regimens. Future research should utilize a dosing regimen that more closely mirrors clinical practice (such as intraoperative loading dose combined with postoperative short-term sedation infusion), and refine the conversion of doses and timing between animal models and clinical settings based on allometric scaling principles, to assess the therapeutic time window and translational potential of this protective effect. Second, pharmacological validation (e.g., SIRT1 inhibitor such as EX-527) is lacking. The combined use of EX-527 or SIRT1 specific agonists (such as SRT1720) in subsequent studies will further strengthen the argument for causality. Third, existing evidence has shown that sirtuin family proteins have clear subcellular localization and functional zoning, which reduces the possibility of direct compensation between each other, but this study did not systematically detect the expression changes of other sirtuins, and cannot completely rule out their potential compensatory effects. Fourth, it has not been determined whether SIRT1 knockdown is independent of propofol in affecting kidney injury. Last but not least, no morphological evidence (e.g., membrane rupture, ballooning) of pyroptosis is provided. The pathological process of AKI involves a complex cell death network, and apoptosis and necrosis may also be involved. It cannot be ruled out that apoptosis and necrosis also occur in this model and are influenced by propofol. In the future, we will explore whether propofol is involved in other cell death modes in AOLT-induced kidney injury, collect relevant data from clinical practice, and explore the possibility of clinical translation.

## Data Availability

The original contributions presented in the study are included in the article/supplementary material. Further inquiries can be directed to the corresponding authors.
